# Metasurface-based ultra-lightweight high-gain off-axis flat parabolic reflectarray for microwave beam collimation/focusing

**DOI:** 10.1038/s41598-019-55221-8

**Published:** 2019-12-12

**Authors:** Sinhara R. Silva, Abdur Rahman, Wilton de Melo Kort-Kamp, Jeremiah J. Rushton, John Singleton, Antoinette J. Taylor, Diego A. R. Dalvit, Hou-Tong Chen, Abul K. Azad

**Affiliations:** 1 Center for Integrated Nanotechnologies, Los Alamos National Laboratory, MS K771, Los Alamos, New Mexico 87545 USA; 2 Physics and Technology Department, Edinboro University of Pennsylvania, Edinboro, PA 16444 USA; 3 Theoretical Division, Los Alamos National Laboratory, MS B262, Los Alamos, NM 87545 USA; 4 Intelligence and Space Research, Los Alamos National Laboratory, MS D448, Los Alamos, NM 87545 USA; 5 Condensed Matter and Magnet Science, Los Alamos National Laboratory, MS E536, Los Alamos, New Mexico 87545 USA; 6 Physical Sciences Directorate, Los Alamos National Laboratory, MS E536, Los Alamos, New Mexico 87545 USA; 7 Theoretical Division, Los Alamos National Laboratory, MS B213, Los Alamos, NM 87545 USA

**Keywords:** Engineering, Materials science, Physics

## Abstract

Ultra-lightweight deployable antennas with high-gain are pivotal communication components for small satellites, which are intrinsically constrained in size, weight, and power. In this work, we design and demonstrate metasurface-based ultra-lightweight flat off-axis reflectarrays for microwave beam collimation and focusing, similar to a parabolic dish-antenna. Our ultra-thin reflectarrays employ resonators of variable sizes to cover the full 2*π* phase range, and are arranged on the metasurface to realize a two-dimensional parabolic focusing phase distribution. We demonstrate a 30° off-axis focusing reflector that exhibits a measured gain of 27.5 dB at the central operating frequency of 11.8 GHz and a 3 dB directionality <$\pm$1.6°. Furthermore, we carry out full-wave simulations of the reflectarray, showing high gain of the beam focusing/collimation functionality, in good agreement with measurements. The demonstrated reflectarrays will enable low-cost, lightweight, and high-gain deployable transceivers for small-satellite platforms.

## Introduction

Small satellites (SmallSats) are emerging space research capabilities for many intriguing applications such as sensing, imaging, tracking, surveillance, and high-speed communications. The use of SmallSats has been rapidly growing in recent years due to multiple factors including miniaturization of electronics, high-speed data processing and high-density data storage, ride-share with other satellites, and reduced cost for their design, build, and operation. Antennas are the key component for satellites to transmit/receive electromagnetic signals. Conventional satellites employ parabolic dish antennas for wireless communication links. Due to the limited size, weight, and power (SWaP) requirements of SmallSats, they mostly rely on small and light-weight monopole, dipole, or helix antennas. Although SmallSats are capable of collecting huge amounts of information, they severely suffer from a bottleneck effect while transmitting/receiving data to the ground/command stations due to the inherit limited performances of these inefficient antennas. Therefore, it is very important to explore alternative approaches to enable lightweight high-gain antennas that occupy a considerably small fraction of the payload and volume of SmallSats. There have been a handful of initiatives in realizing high-performance lightweight antennas for small satellites^1–3^ including Fabry-Pérot cavity antennas^4^, deployable mesh reflectors^5,6^, inflatable balloon reflectors^7^, deployable waveguide slot antennas^8^, and reflectarrays^9^.

The working principle of a reflectarray antenna is similar to that of a reflecting dish antenna, consisting of a feed and a reflecting surface that combined produce a prescribed secondary radiation pattern^10^. Unlike a reflecting parabolic dish, the reflectarray is generally flat and consists of an array of reflecting elements that are arranged on a surface to produce a desired phase distribution. The concept of reflectarray was first demonstrated using an array of short-circuited square waveguides and the desired phase profile was obtained by adjusting the length of each waveguide in the array^11^. Since the first demonstration, there have been many efforts to increase the performance of reflectarrays by implementing various types of phase manipulating elements, including variable delay lines^12^, frequency selective surfaces^13^, and metasurfaces^14,15^. Many of these demonstrations are focused on enabling broadband and polarization independent radiation at the price of using multi-layers^16,17^ or thick substrates^18^, making them bulky for small satellite applications. Very recently, planar reflectarrays have been studied as a viable option for enabling high-gain antennas for SmallSat applications^19,20^. While these results are encouraging, further studies are required for addressing issues like phase quantization errors, estimation of focal depth, and reduced stowage volume and payload. Most metasurface reflectarrays employ variable sized metallic square patches as the unit cell, which show high sensitivity to phase changes with the length of the resonators around the resonant length, making it difficult to fabricate patches with great precision in order to obtain the required phase and reduce phase quantization errors.

## Results

In this work, we introduce a new resonator design that consists of a pair of identical rectangular patches. The gap between the two patches, along with their variable length and width, allow additional phase tuning, and therefore reduce phase quantization errors. We demonstrate a 30° off-axis focusing/collimation flat reflectarray (total size 44 cm × 44 cm based on a single layer metasurface separated from a metallic ground plane by a thin dielectric spacer. Our polarization-dependent ultrathin reflectarray operates at a frequency of 11.8 GHz, has a focal length of $57\,{\rm{cm}}$, and shows excellent beam focusing/collimation properties with a measured gain >27 dB at the focal point. The metasurface reflectarray has a 6 cm-long depth of the focus that makes it more robust against misalignment than traditional parabolic dish antennas, and possesses an overall areal weight density of 0.11 g/cm^2^ making it suitable for SmallSat platforms.

### Design of the reflectarray

**Figure 1:**
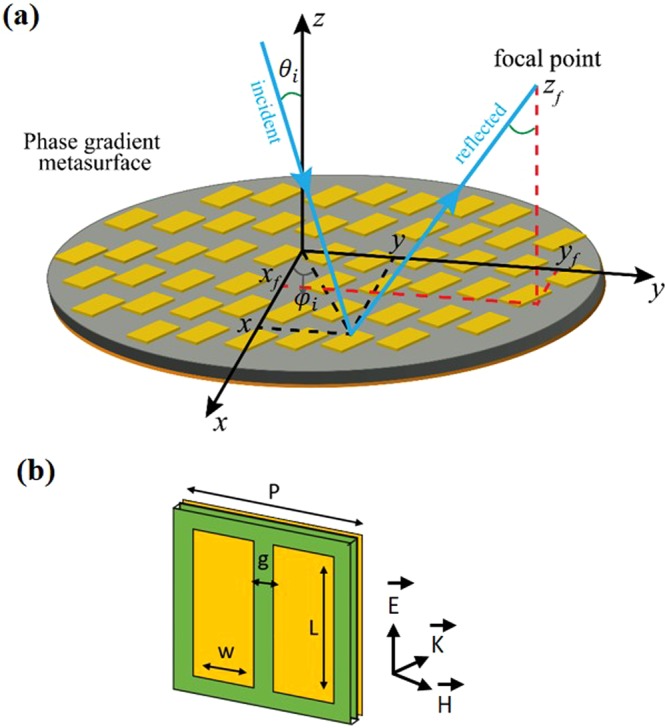
(**a**) Schematic of an off-axis focusing phase gradient metasurface reflectarray. For a plane wave with incidence angle $\,{\theta }_{i}$ and focal point *(x*_*f*_, *y*_*f*_* , z*_*f*_), the required phase distribution $\psi (x,y)\,$is given by Eq. (1). (**b**) Unit-cell design, consisting of a pair of rectangular patch resonators (copper) with their longer axis along the electric field of the microwave radiation and separated from a copper ground plane by a thin dielectric spacer.

Let us first recall the analytical expression for the required phase distribution of a collimating/focusing reflectarray for an arbitrary angle of incidence/emittance and generic focal position. The schematic of such reflectarray is shown in Fig. 1(a), where *θ*_*i*_ is the polar angle of the incident/emitted collimated beam with respect to the surface normal *z*-axis and (*x*_*f*_*, y*_*f*_ , *z*_*f*_) are the focal point coordinates. The required phase distribution as a function of position (*x*, *y*) on the metasurface is given by

1 $$\psi (x,y)=\frac{2\pi }{\lambda }\{\,\sqrt{{(x-{x}_{f})}^{2}+{(y-{y}_{f})}^{2}+{{z}_{f}}^{2}}-{z}_{f}+sin\,{\theta }_{i}\,[(x-{x}_{f})\,cos{\psi }_{i}+(y-{y}_{f})\,sin\,{\psi }_{i}]\},$$

where *λ* is the operating wavelength, ${\psi }_{i}$ is the azimuthal angle of the incident/emitted collimated beam, and $f=\sqrt{{{x}_{f}}^{2}+{{y}_{f}}^{2}+\,{{z}_{f}}^{2}}$ is the focal length. In order to achieve focusing for normal incidence $\,({\theta }_{i}=0)$, the phase profile that should be imparted by the metasurface simplifies to $\psi (x,y)=\frac{2\pi }{\lambda }\{\,\sqrt{{(x-{x}_{f})}^{2}+{(y-{y}_{f})}^{2}+\,{{z}_{f}}^{2}}\,-{z}_{f}]\}.$For ${x}_{f}=\,{y}_{f}=0,\,$ this equation gives the conventional formula for an on-axis focusing lens or a center-fed dish antenna^21^. In order to avoid the intrinsic problems of center-fed parabolic dishes, which include feed blockage and shadowing^22^, in this work we demonstrate an off-axis focusing metasurface reflectarray. Unlike a center-fed reflector or lens, where the phase distribution is radially symmetric, the phase distribution of an off-axis parabolic reflectarray is asymmetric.

Focusing/collimation requires a full 2π phase tunability, and for standard optical components the desired phase is obtained via propagation (reflection) through (from) specifically shaped materials, *e.g*., the thickness of the lenses or curvature of reflectors show a continuous spatial phase modulation. In metasurfaces, which are often realized as 2D periodic arrays, the phase distribution is spatially discretized and the phase remains constant within the period of the unit cell. The scattering phase and amplitude of the metasurface unit depend on the judicious choice of the unit cell design. A reflectarray metasurface requires a unit cell with high reflectance while providing a 2π phase dispersion. For our proposed metasurface, we employ a square unit-cell design consisting of a pair of rectangular patch copper resonators separated from a copper ground plane by a thin dielectric spacer, as shown in Fig. 1(b). Our design is polarization-dependent, with the electric field of the incident/emitted microwave radiation along the longer axis of the patches. The pixel size of the reflectarray is defined by the unit-cell size, which needs to be small to minimize the pixelation effects while providing the desired phase and amplitude. We use a unit-cell of lateral size of 10 mm to operate around 12 GHz, which introduces ~2000 pixels in our $44\,{\rm{cm}}\,\times 44\,{\rm{cm}}$ reflectarray surface.

**Figure 2:**
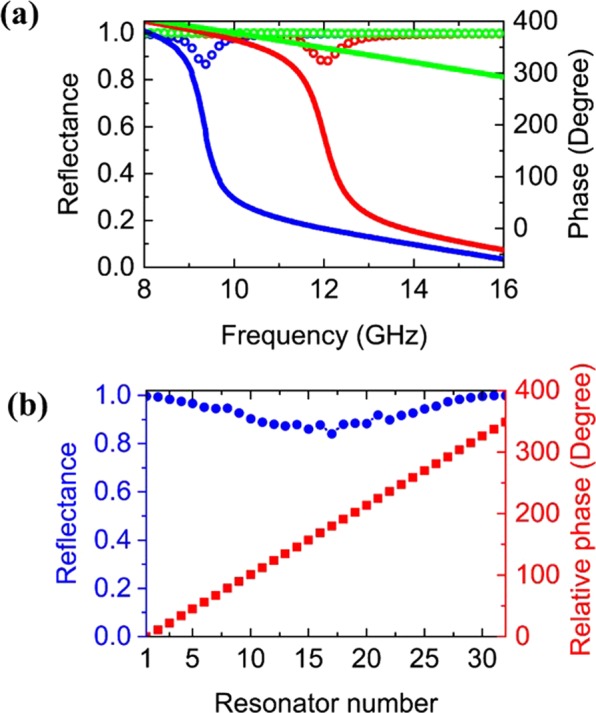
(**a**) Simulated reflection phase (solid curves) and amplitude (dotted curves) as a function of frequency for three different resonator unit cells. The structural parameters of resonator #1 (blue) are L_1_ = 8.0 mm, w_1_ = 3.0 mm, g_1_ = 1.0 mm, resonator #17 (red) are L_17_ = 6.25 mm, w_17_ = 2.875 mm, g_17_ = 0.5 mm, and resonator #32 (green) are L_32_ = 2.5 mm, w_32_ = 2.75 mm, g_32_ = 0.5 mm. Choosing the phase of resonator #1 as 0° at 12 GHz, the relative phases for resonators #17 and #32 are 180° and 348.75° at the same frequency (L, w and g parameters for all 32 resonators are given in the supplementary material). (**b**) Phase distribution of the 32 unit cells of the reflectarray panel, which indicates the discretization of the phase into a finite number of elements (red). The blue curve shows the corresponding simulated reflectance.

To cover the required full 2π phase range, we vary the structural parameters (length L, width w, and gap g) of the resonators and obtain 32 phase values from 0 to 2π in increments of $\pi$/16. The discrete values of the phase are derived using commercially available full-wave frequency-domain numerical simulation software (CST). We assume the local phase approximation, and apply periodic boundary conditions to obtain the reflection phase and amplitude for various combinations of the geometric parameters L, w, g with realistic material properties (copper conductivity of 5.8 × 10^7^S/m and the dielectric constant of the spacer is 3.55 with a loss tangent of 0.0027). The thicknesses of the resonators and spacer are 17 µm and 0.508 mm, respectively. For each resonator, we run a set of simulations to achieve the desired phase while minimizing reflection loss. Figure 2(a) shows three sets of phase and amplitude dispersion curves for three resonator designs that provide a relative phase difference of 0°, 180°, and 348.75° with their corresponding reflectance higher than 85%. The incident collimated microwave initiates a resonant current in the patches, which then interacts with the image current provided by the ground plane, and the nearfield coupling of these currents results in a broad phase response of the reflected field without polarization conversion^23,24^. The simulated phase and corresponding reflectance of resonators 1 to 32 are shown in Fig. 2(b). Digitization of the 2*π* phase into pre-selected and fixed number of elements is convenient because these elements can be reused for designing other reflectarrays for different $\,{\theta }_{i}$, $\,{\varphi }_{i}$, and *f* working at the same operating frequency.

### Fabrication and characterization of the reflectarray

**Figure 3:**
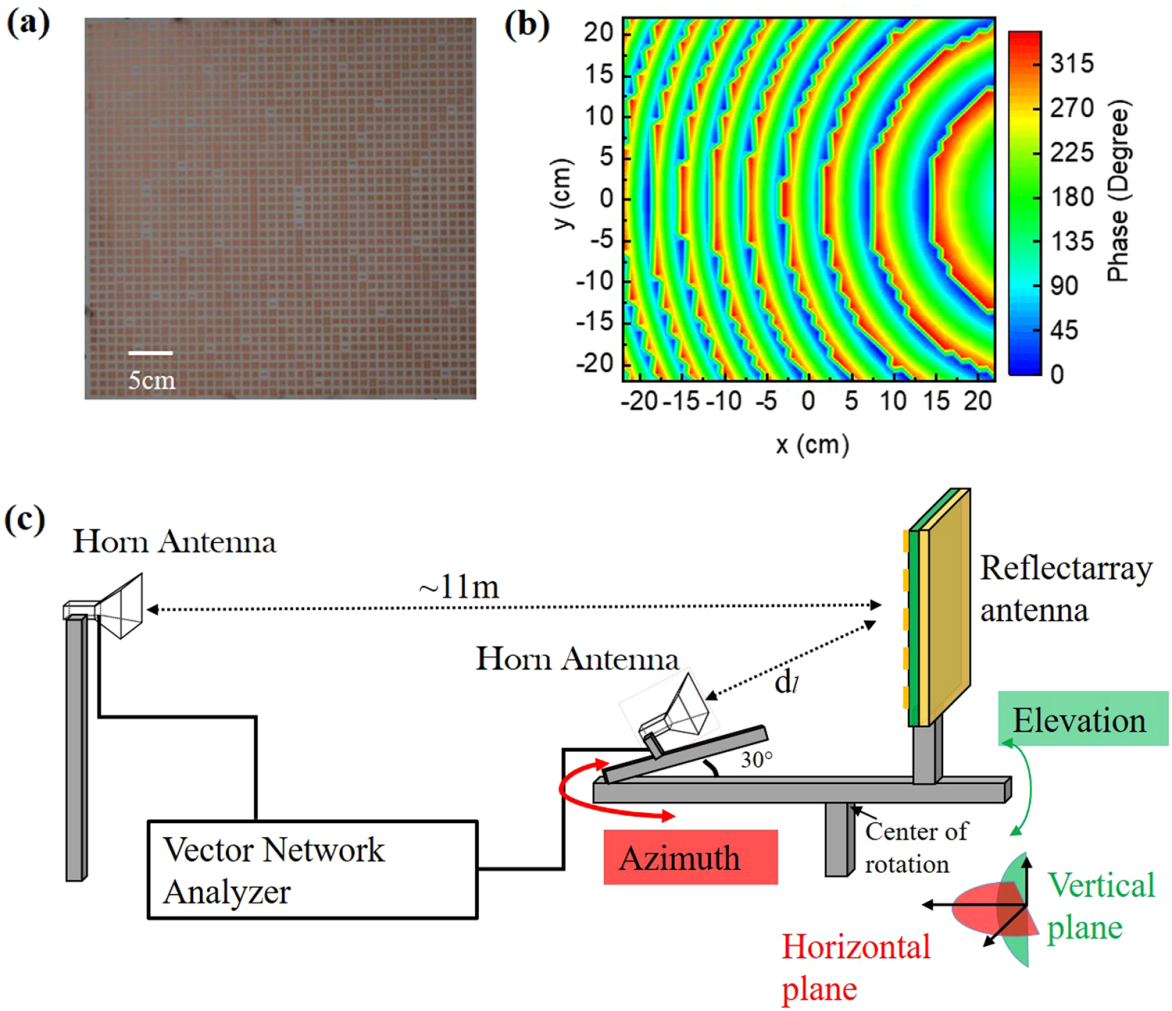
(**a**) Photograph of the fabricated reflectarray metasurface. (**b**) Spatial phase profile of the metasurface, where the color coding represents the use of the different 32 discrete phase values listed in Fig. 2(b). (**c**) A schematic of the experimental setup for characterizing the off-axis metasurface.

The metasurface reflectarray was fabricated on a double copper cladding printed circuit board (PCB) from Rogers Corporation (RO 4003C) using chemical etching of copper. The weight of the fabricated reflectarray is 220 g (0.11 g/cm^2^). An image of the fabricated reflectarray is shown in Fig. 3(a). Figure 3(b) shows the spatial phase profile of the metasurface, where the color coding represents the use of 32 discrete phase values shown in Fig. 2(b). The fabricated reflectarray metasurface was characterized inside an anechoic chamber using a pair of broadband horn antennas (SAS-571) and a vector network analyzer (Agilent N5230A). The feed horn antenna (transmitter, Tx) was placed along the 30° off-axis line at a distance *d*_1_ = 58 cm (designed focal length) from the reflectarray, and the receiving horn antenna (detector, Rx) was placed ~11 m (far field) away from the reflectarray antenna. The schematic of the experimental setup is shown in Fig. 3(c). In order to simplify the experimental setup, we choose the off-axis focal point to lie on the *yz*-plane (i.e., ${x}_{f}=0$). The reflectarray and the feed-horn are placed on a computer-controlled gimbal, which can rotate the direction of the collimated microwave output beam along a horizontal axis (azimuth) and along a vertical axis (elevation) as indicated by the arrows. Furthermore, the separation *d*_1_ between the reflectarray and Tx along the focal line can be varied mechanically. Although we designed the reflectarray to operate at 12 GHz, the measurements show the highest gain at 11.8 GHz. During the phase simulations using CST, we considered a reflectarray consisting of an infinite array of resonators of same size. However, in a focusing/collimating reflectarray, any given resonator does not have identical nearest neighbors, so that the used local phase approximation may be responsible for the frequency discrepancy between the design and measurements. The measured distance of the focusing center is observed at a distance *d*_1_=57 cm, which is a little shorter than our designed focal length of 58 cm. The disagreement between the designed focal length and the measurement may be due to the paraxial ray approximation in designing the reflectarray.

**Figure 4:**
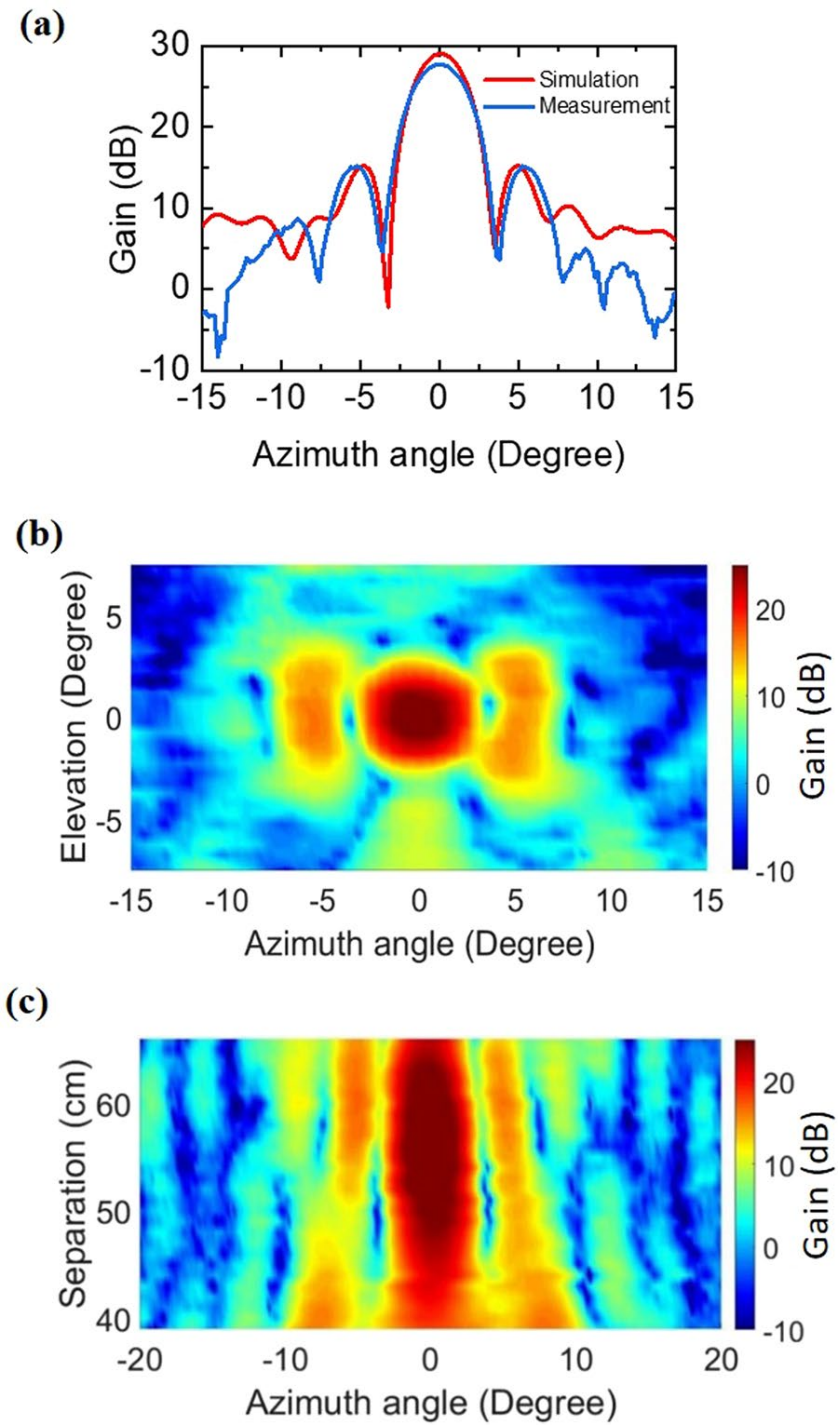
(**a**) Measured (blue) and simulated (red) gain of microwave radiation of the reflectarray as a function of the azimuth scan angle. The elevation angle is 0° and *d*_1_ = 57 cm. (**b**) Gain profile as a function of the elevation and azimuth angles at the focus (*d*_1_ = 57 cm), and **(c**) as a function of azimuth angle and separation *d*_1_.

Figure 4(a) depicts the measured azimuth angle dependent gain at frequency 11.8 GHz for the Tx position at d_1_ $=57\,{\rm{cm}}$ for a fixed elevation angle of 0°. A net gain of 27.5 dB is observed for an azimuth angle of zero degree, confirming the validation of our reflectarray design for beam focusing/collimation. To verify our experimental results we carried out a full wave simulation for the reflectarray using HFSS, shown in Fig. 4(a), which does not employ any local phase approximation. The simulation results agree well with the experimental data, but shows a small deviation (<0.25°) at the 1^st^ side lobe position probably due to fabrication tolerance. The measured gain shows a characteristic angle dependence with a main lobe along the *z*-axis followed by side lobes as the azimuth angle varies in both positive and negative directions. The first minimum appears at ±3° and the first side lobes are located at ±5°. We observe higher than 12 dB contrast between the main and the first side lobes, which is primarily due to the finite size of the reflectarray and can be enhanced by increasing the array size and optimizing the design. We further investigated the far-field radiation pattern as a function of the azimuth and elevation angles, see Fig. 4(b). The radiation pattern shows a dominant Gaussian type main lobe, which is symmetrically bounded within ±3° for both azimuth and elevation angles. The angle for 3 dB gain enhancement is less than 1.6°, confirming good directionality and beam collimation. The circular field distribution is also a manifestation of good beam collimation, a pre-cursor for long-haul communication link. Finally, to demonstrate beam focusing, we measured the gain by varying the separation *d*_1_ between the reflectarray and Tx along the focal line from *d*_1_ = 39 cm to *d*_1_ = 66 cm in steps of 1 cm. For each position of Tx, we changed the azimuth angle from −15° to +15° in the horizontal direction while keeping the elevation angle fixed to 0°. Fig. 4(c) shows a surface plot of the measured gain for a quasi-plane wave incident beam as a function of azimuth angle and distance to the image plane *d*_1_. The maximum gain was found to extend from *d*_1_ = 53 cm to *d*_1_ = 59 cm, i.e., a focusing range of 6 cm. Beyond this range, the gain rolls-off gradually, confirming the focusing behavior. The 6 cm focusing range may be beneficial for a deployable antenna where post deployment fine adjustment is not possible.

Larger off-axis focusing can be achieved using our resonators that span a 0–2π phase coverage. To this end, once the desired focal point is chosen and the required two-dimensional phase distribution is determined according to Eq. (1), we would need to re-arrange the distribution of the resonators in order to provide the required phase distribution. We would like to note, however, that for a given focal length the gain at the focal point is maximum for on-axis (${\theta }_{i}$ = 0°) focusing, and decreases monotonically as the angle increases (*i.e*., as the focal point gets closer to the metasurface plane). On-axis focusing has the problem of interference and shadowing due to the interaction between the detector and the metasurface, as mentioned above. Off-axis focusing at 90° (grazing focusing) is not experimentally feasible. For this, all resonators on the metasurface should re-direct the normally incident beam into the grazing direction, and this will result in cross-talk between resonators that will degrade any focusing effect. Finally, we would like to note that the reflectarray is a reciprocal optical component. Therefore, if one uses the Rx as the transmitter rather than as a receiver, then the transmitted collimated microwave beam can be detected at Tx, which now acts as the receiver rather than as a transmitter.

## Conclusion

In conclusion, we have designed, simulated, fabricated, and characterized a metasurface-based reflectarray antenna at microwave frequencies consisting of a resonator array with off-axis focusing/collimation. The fabricated reflectarray demonstrates excellent focusing and collimation of the radiated microwaves from a feed-horn antenna. The measured results show a gain as high as 27.5 dB, and agree well with the designed focal length and off-axis angle.

## Supplementary information

supplementary information
