# Supplementary material for: Metasurface-based ultra-lightweight high-gain off-axis flat parabolic reflectarray for microwave beam collimation/focusing
Source: Sci Rep. 2019 Dec 12;9:18984. doi: 10.1038/s41598-019-55221-8 (PMC6908700; doi:10.1038/s41598-019-55221-8)
Supplement: Supplementary file 1 — supplementary information [file 41598_2019_55221_MOESM1_ESM.docx]

**upplementary material**

Design parameters of 32 resonators.


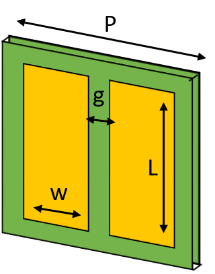


| Resonator number | Phase(Degree) | g(mm) | L(mm) | w(mm) |
| --- | --- | --- | --- | --- |
| 1 | 0 | 1 | 8 | 3 |
| 2 | 11.25 | 1 | 7.25 | 3.5 |
| 3 | 22.5 | 1 | 6.875 | 3 |
| 4 | 33.75 | 0.75 | 6.625 | 3.25 |
| 5 | 45 | 1 | 6.625 | 3 |
| 6 | 56.25 | 0.5 | 6.5 | 2.75 |
| 7 | 67.5 | 0.75 | 6.5 | 2.875 |
| 8 | 78.75 | 0.75 | 6.5 | 2.625 |
| 9 | 90 | 0.5 | 6.375 | 3.125 |
| 10 | 101.25 | 0.5 | 6.375 | 3 |
| 11 | 112.5 | 1 | 6.375 | 3 |
| 12 | 123.75 | 0.5 | 6.375 | 2.75 |
| 13 | 135 | 0.75 | 6.375 | 2.625 |
| 14 | 146.25 | 0.5 | 6.25 | 3.25 |
| 15 | 157.5 | 1 | 6.375 | 2.5 |
| 16 | 168.75 | 1 | 6.25 | 3.25 |
| 17 | 180 | 0.5 | 6.25 | 2.875 |
| 18 | 191.25 | 1 | 6.25 | 3 |
| 19 | 202.5 | 0.75 | 6.25 | 2.75 |
| 20 | 213.75 | 0.75 | 6.25 | 2.625 |
| 21 | 225 | 1 | 6 | 3.5 |
| 22 | 236.25 | 1 | 6.25 | 2.5 |
| 23 | 247.5 | 1 | 6.25 | 2.25 |
| 24 | 258.75 | 0.75 | 6.125 | 2.75 |
| 25 | 270 | 0.5 | 6.125 | 3 |
| 26 | 281.25 | 0.5 | 5.875 | 3.25 |
| 27 | 292.5 | 0.5 | 5.875 | 2.875 |
| 28 | 303.75 | 1 | 5.75 | 3 |
| 29 | 315 | 1 | 5.625 | 2.75 |
| 30 | 326.25 | 1 | 5 | 3 |
| 31 | 337.5 | 0.75 | 3 | 2.75 |
| 32 | 348.75 | 0.5 | 2.5 | 2.75 |
